# The Relation of the Specific Gravity of the Blood to Its Percentage of Hemoglobin

**Published:** 1900-10

**Authors:** F. C. Busch, A. T. Kerr, F. Filsinger

**Affiliations:** Buffalo, N. Y.; From the Pathological Laboratory, University of Buffalo, 145 Allen Street; Buffalo, N. Y.; From the Pathological Laboratory, University of Buffalo, 145 Allen Street; Buffalo, N. Y.; From the Pathological Laboratory, University of Buffalo, 145 Allen Street


### BUFFALO MEDICAL JOURNAL.

VOL. XL.—LVI. OCTOBER, 1900. No. 3.

### Original Communications.

### THE RELATION OF THE SPECIFIC GRAVITY OF THE BLOOD TO ITS PERCENTAGE OF HEMOGLOBIN.

BY F. C. BUSCH, B. S., M.D., A. T. KERR, B.S., M.D., and F. FILSINGER, M.D., of Buffalo, N. Y.

From the Pathological Laboratory, University of Buffalo.

SINCE the publication of our paper in the *Medical News* of December, 1895,[1] we have added a number of new cases to our list of observations. In addition to these, we have included in the present paper much of the description of methods given in our former paper, together with the results of our first observations.

For the determination of the hemoglobin in our first series we used v. Fleischl's hemometer and Gowers's hemoglobinometer. We made no count of the blood corpuscles, in comparison, but it has been found by Jones, Hammerschlag, Schmaltz and others, that the specific gravity corresponds more closely with the amount of hemoglobin than with the number of red blood-corpuscles.

For determining the specific gravity of the blood, a variety of methods have been used. A good method, but one which takes considerable time, is that with the pycnometer. This, as used by Schmaltz, is a capillary tube of 0.1 cc.m capacity, which is first weighed empty, then when filled with water, and, finally, when filled with blood. The weight of the tube is subtracted and the specific gravity calculated from the weight of the blood as compared with that of the water.

Fano, in 1882, used a method which depends upon the principle that a body when immersed in a fluid will float indifferently in that fluid when the specific gravities of the two are the same. The fluid used, was a solution of gum in water. To make this heavier, he

1. Busch and Kerr: "The Relation Between the Specific Gravity of the Blood and its Hemoglobin Percentage," *Medical News*, December 21, 1895."

added a denser gum solution. To make it lighter he added water. Blood was introduced into this and the liquid made to correspond in specific gravity to that of the blood. The specific gravity of the liquid was then determined and thus at the same time that of the blood.

Roy, in 1884, devised a method applying this same principle. He used solutions of salt or other suitable substances ranging in specific gravity from 1.035 to 1.075. For making the test he used a modified hypodermic syringe. This had a small steel tube prolonged inward from the tip so as to be seen through the glass sides of the barrel. The syringe was filled with a salt solution and a drop of blood drawn into it. As it emerged from the steel tube into the solution he noted whether it rose or sank. If it sank, a new solution of higher specific gravity was chosen; if it rose, one of lower specific gravity was taken. Finally, one was obtained in which the blood neither rose nor sank, or two were found in one of which it rose and in the other sank. In the latter case the specific gravity of the blood was between the two.

Roy's method as modified by Jones, is as follows: The apparatus consists of from 20 to 25 one-ounce glass bottles filled with standard solution of glycerin and water, differing one from the other by 0.001 specific gravity and ranging from 1.027 to 1.075; a number of fine glass pipettes drawn out to a point and bent at right angles near the tip; a cylindrical glass jar of about one-dram capacity; and a number of clean suture-needles. By means of the pipette the blood is introduced into the one-dram jar filled with one of the standard solutions chosen by guess from the appearance of the patient. It is then blown out gently, being given an impetus in a horizontal direction owing to the bent tip of the pipette. If it rises or sinks other solutions are chosen, as in Roy's method.

Landois used solutions of sodium sulphate ranging between 1.050 and 1.070.

Siegel has adopted a device whereby the frequent standardisation with the Jones-Roy method is obviated. He covers the surface of his standard solutions with a layer of olive oil, introducing the blood through this by means of a glass-tube covered by a rubber cap.

In our determinations we have used Hammerschlag's method. The necessary apparatus consists of a urinometer jar, a urinometer, a pipette of small caliber, a glass rod, some fine steel pens, a bottle of chloroform, one of benzol, and a mixture of the two. The finger, after being washed with bichloride and alcohol, is punctured. A good sized drop of blood is introduced into the chloroform-benzol mixture. If the drop sinks the mixture must be made heavier by adding chloroform. If it rises, the mixture is too heavy and must be made lighter by adding benzol.

It is desirable not to divide the drop of blood into several, but on the other hand, care must be taken to get the chloroform and benzol thoroughly mixed by stiring with the glass rod. In order to obtain this end it is better, when the liquid is too heavy, to add an excess of benzol and then obtain the required density by adding chloroform, slowly and carefully until the drop of blood floats indifferently in the mixture. When this occurs, the drop of blood is of the same specific gravity as the mixture, and by determining that of the mixture with a urinometer, we have also determined that of the blood.

We have found it most convenient to obtain the blood from the middle finger of the left hand, making the puncture to the side of the tip on the palmer surface. For this purpose, we have used an ordinary sharp-pointed steel pen, with one nib broken off. The pen was sterilised by heat and a new one used for every test. The blood was drawn up into a pipette of fine caliber, which was introduced into the middle of the chloroform-benzol mixture and nearly all of the blood gently blown out. We were careful, however, not to blow all of the blood out, but to leave a little in the tip of the pipette, shaking off the adhering drop. In this way the error of mixing air with the blood was avoided.

Ziegelroth[3] claims it is better to obtain the blood from the lobe of the ear, since it is nearer the density of the blood in general.

The same chloroform-benzol mixture may be used repeatedly, if filtered after each test. The urinometer jar must be scrupulously clean; otherwise some fine particles of dust might adhere to the drop of blood and cause an error.

It is also imperative to take the reading as soon as possible after a satisfactory mixture is obtained; since there is considerable danger especially in a warm room of error through vaporisation of the volatile fluid. Besides this, the globule of blood, after a little time appears to shrink and change its shape.

The hemoglobinometer of Gowers is usually manufactured with but one colored tube which is for use with daylight only. There is another form in which there are two tubes, one for use with daylight and the other with artificial light. The one we have employed is of the former kind. In making the comparison with it, one must hold the instrument against a white background, opposite the source of light, or as recommended by Landois, between the eye and the light.

In the use of v. Fleischl's hemometer, artificial light is necessary, and the test must be made in a room from which daylight is excluded. We have for the sake of convenience adapted a simple device, allowing us to carry our dark room with us. A piece of card-board is cut so as to fit over the top of the canvas traveling bag in which we carry our instruments. The hemometer is placed in one end of the case, and a small lamp in the other. A hole is cut in the card board to give passage to the lamp chimney. Another small hole is cut so as to be directly over the well of the instrument. At the side of this hole, a window, closed by a card-board flap, is cut, for the introduction of the hand, in order to move the wedge of the instrument.

The specific gravity in health has been differently given by different observers.

| MALES. | | FEMALES. | |
|---|---|---|---|
| Bennett | 1.055–59 | Perrier | 1,051–55 |
| Perrier | 1.056–59 | Davy | 1,045–56 |
| Frenn | 1.062 | Nasse | 1 055–55 |
| Davy | 1.052–60 | Becqurel and Rodier | 1,054–60 |
| Nasse | 1.055–59 | Schmidt | 1,050 |
| Becqurel and Rodier | 1.058–60 | Quincke | 1,058–60 |
| Muschenbroeck | 1.054 | Jones (about) | 1,055 |
| Denis | 1 059 | Schmaltz | 1,056 |
| Schmidt | 1.060 | Becker | 1,057–56 |
| Loyd Jones | 1.058 | Landois | 1,057–55 |
| Schmaltz | 1.059 | | |
| Becker | 1.054–60 | | |
| Landois | 1.056–50 | | |

Hammerschlag found the specific gravity of healthy males—between the age of 20 and 40 to vary from 1.057 to 1.625 and in females from 1.0535 to 1.061.

In the tropics, the average for normal adults, according to Glogner, is 1053.6, and according to Eijkman, 1.057.4.

The average specific gravity of the blood in the newborn is 1.060; for the two to four months old child it is 1057; for the twelve months old child it is 1050; and from the second to the tenth year it is 1052. Monti[24].

Jones states that venous blood is denser than arterial. Sherrington and Copeman found almost no difference in density between venous and arterial blood. In passive congestion, as in a ligatured finger, they agree that there is an immediate rise in specific gravity. This rise vanishes immediately upon the disappearance of the congestion. A characteristic property of the blood is to keep its specific gravity constant. Thus, after drinking large amounts of fluid, the change in specific gravity disappears in from one-half to one hour. Sherrington and Copeman found that after injecting large quantities of salt solution into the veins of rabbits, the specific gravity of the blood returned to the normal in a very short time. It was discovered by Hoch and Schlesinger that a marked change in the specific gravity of the blood as a whole might take place without affecting that of the serum. Therefore it appears that, usually, the principal factors in a change of specific gravity of the blood from the normal are the cellular constituents.

According to Ziegelroth[34], the specific gravity of the blood and of the body as a whole are nearly the same; that of the former being on the average, 1.057 and of the latter, 1.055. The specific gravity of the blood is much more stable than that of the body, as a whole. Copious sweating, he found, had no influence on the specific gravity of the blood.

Of the solid constituents of the blood, in man, the hemoglobin constitutes about 60 per cent. (Hoppe-Seyler). In females it is somewhat less. This being the case, variations in the percentage of hemoglobin must immediately affect the specific gravity of the blood. Hammerschlag maintains that it is therefore possible to determine from the specific gravity, accurately enough for clinical purposes, the hemoglobin percentage for a given sample of blood and has formulated the following table of specific gravities with hemoglobin equivalents.

| Specific Gravity. | Hemoglobin, Per cent. | Specific Gravity. | Hemoglobin. Per cent. |
|---|---|---|---|
| 1.033–1.035 | 25–30 | 1.048–1.050 | 55–65 |
| 1.035–1 038 | 30–35 | 1.050–1.053 | 65–70 |
| 1.038–1.040 | 35–40 | 1 053–1.055 | 70–75 |
| 1.040–1.045 | 40–45 | 1.055–1.057 | 75–85 |
| 1.045–1 048 | 45–55 | 1.057–1.060 | 85–95 |

According to Hammerschlag, this table holds best for cases of anemia, including chlorosis, tuberculosis and malignant tumors. But in interstitial nephritis, the specific gravity is relatively lower than the hemoglobin. In circulatory disturbances, even when edema is present, the specific gravity is generally normal. In fever, it is relatively lower than the hemoglobin, rising after the fall of the fever. According to Monti[24], a normal specific gravity occurred with a diminution of hemoglobin in chlorosis, mild anemia, heart lesions with anemia and in tuberculosis with evening temperature; a higher specific gravity and normal hemoglobin in developing acute fevers; a high specific gravity and diminished hemoglobin in acute and chronic diseases, of long duration; a lower specific gravity and normal hemoglobin in acute nephritis with dropsy; a low specific gravity and lower hemoglobin per cent. in leukemia and pernicious anemia; a low specific gravity and a much lower hemoglobin percentage in severe anemias and chlorosis.

Out of the 100 consecutive cases taken for comparison of the three methods (v. Fleischl, Gowers and specific gravity) in our series of 1895, the amount of hemoglobin estimated from the specific gravity and as determined by the Fleischl method differed by less than 5 per cent. in 24 cases: between 5 per cent. and 10 per cent. in 24 cases: between 10 per cent. and 20 per cent. in 26 cases; and more than 20 per cent. in 26 cases. The specific gravity method ran below the Fleischl in but 15 cases and in 5 of these less than 5 per cent. Therefore the difference observed usually showed the readings higher by the specific gravity method than by the Fleischl instrument.

In the same cases the readings of Gowers's apparatus differed by less than 5 per cent from Fleischl's in 36 cases; between 5 per cent. and 10 per cent. in 21 cases; between 10 per cent. and 20 per cent. in 33 cases; and by more than 20 per cent. in 10 cases. As a general rule they were higher than with Fleischl's, but not quite as high as those estimated from the specific gravity. Comparing the percentage obtained from the specific gravity with the Gowers's readings, in 33 cases there was less than 5 per cent. difference; in 31 cases the difference was between 5 per cent. and 10 per cent.; in 26 cases between 10 per cent. and 20 per cent.; and more than 20 per cent. in 10 cases.

From the foregoing comparison, it appears that in approximately one-half of our cases the hemoglobin as determined from the specific gravity corresponded quite well with the hemoglobin as determined by the Fleischl hemometer; that this correspondence was fair between the specific-gravity determinations and the Gowers determinations in more than one-half of our cases; that there was a somewhat closer correspondence between the determinations from the specific gravity and the Gowers than there was between the Gowers and Fleischl instruments; that the Fleischl instrument gave relatively lower readings.

The differences in the readings of the three methods may be due: (1) to errors in the specific gravity method; (2) to the inaccuracy of the table giving the hemoglobin equivalent for the specific gravity; (3) to the errors of the v. Fleischl and Gowers instruments.

We are convinced that both v. Fleischl's and Gowers's instruments, particularly the latter, are liable to err to a considerable degree.

Osler says that the error in the Fleischl instrument may not be more than 2 per cent. in blood which is nearly normal, but he cites Neubert and Letzius as having shown that in a much impoverished blood the error may be as high as 20 per cent. In using two Fleischl instruments in comparison in the same cases, we have generally found a difference in reading between the two. In 30 per cent. of these comparisons the difference was as much as 10 per cent. We have also found that in about one-fifth of our cases we disagree in our readings of the same instrument; 40 per cent. of these differences were more than 5 per cent.

We feel some hesitancy in stating that in consecutive tests, made with the same Fleischl instrument, in the same cases, within a few minutes, we have found differences in readings of as much as 10 per cent. This occurred to us after having used the instrument almost daily for nearly a year, and after having used it many hundred times. Such errors can only be explained by acknowledging inaccuracy in technic, but a method so easily liable to such serious errors could hardly be relied upon for more precise results in the hurry of ordinary clinical work.

In the same series of consecutive tests, the specific gravity and the hemoglobin percentage as determined by it, varied to an insignificant degree. The hemoglobin percentages, as determined by Gowers's instrument, in some of the tests, varied as much as 10 per cent. In our experience with the Gowers instrument, we have found it very unsatisfactory. It is often quite impossible to get the tint of the diluted blood to correspond with that of the standard 1 per cent. solution. Even when this is attained, a difference in shade may be produced by looking at the instrument somewhat from the side instead of from straight in front, by holding the paper for reflection farther away from or nearer the instrument, by holding the instrument between the eye and the window, or by moving farther away from the window. In the last case, in a number of instances, the readings obtained by moving 20 feet away from the window became as much as 15 per cent. higher. Therefore it would seem that on a cloudy day readings would be higher than on a bright day. The differences in readings between holding the instrument against a white background and holding it directly between the eye and the light were as much as 10 per cent.[1]

As may be seen from the foregoing comparisons and the observations of others the Fleischl instrument is not entirely reliable; there-

1. See Limbeck, pp. 18, 19, for analogous observations.

fore Hammerschlag's table, which is based upon this instrument, must be inaccurate. Taking all these conditions into consideration, there appears to be quite a constant correspondence between the specific gravity and the percentage of hemoglobin, and we think that, with a table based upon a much larger number of cases and a more reliable estimation of the amount of hemoglobin, this method would be as accurate for clinical purposes as the methods now in vogue, and possibly more so.

Cora Lichty, *Philadelphia Med. Jour.*, 1898, ii., 242-44, gives the following table of specific gravity and hemoglobin equivalents.

| Specific Gravity. Hammerschlag's Method. | Hemoglobin Per cent. v. Fleischl? | Specific Gravity. Hammerschlag's Method. | Hemoglobin Per cent. v. Fleischl? |
|---|---|---|---|
| 1035–38 | 25–30 | 52–54 | 65–70 |
| 38–43 | 30–40 | 54–56 | 70–75 |
| 43–45 | 40-45 | 56–60 | 75–85 |
| 45–47 | 45–50 | 60–63 | 85–95 |
| 47–49 | 50–55 | 63–65 | 95–100 |
| 49–52 | 55–65 | | |

Yarrow and Hitchens[32] have formulated the following table, using standard solutions of hemoglobin for comparison:

| Specific Gravity. | Hemoglobin Per cent. | Specific Gravity. | Hemoglobin Per cent. |
|---|---|---|---|
| 1030 | 20 | 1051 | 65 |
| 1035 | 30 | 1052 | 70 |
| 1041 | 40 | 1053.5 | 75 |
| 1042.5 | 45 | 1056 | 80 |
| 1045.5 | 50 | 1057.5 | 90 |
| 1048 | 55 | 1059 | 100 |
| 1049 | 60 | | |

They also strongly recommend because of its accuracy Oliver's tintometer for determining the hemoglobin.

In the following table we have recorded a new series of cases. All of these with the exception of 21, which were kindly placed at our disposal by Dr. Albert E. Woehnert, were made at the Erie County Hospital, and we are indebted to Dr. C. R. Orr for a portion of these records.

In the thirty-one records of persons in apparent health, the greatest difference between the hemoglobin by the v. Fleischl instrument and that estimated from the specific gravity was 19 per cent. The discrepancy in this case was probably due to error in the v. Fleischl reading, since the estimated hemoglobin corresponded very closely to the number of red corpuscles.

In eleven cases there was practically no difference between the estimated and determined hemoglobin. In fifteen of the remaining thirty-one cases the difference was less than 10 per cent. In the simple anemias the correspondence between the two was, as a rule, very close. One case, however, showed a difference of 15 per cent., the determined hemoglobin being higher than that indicated by the specific gravity.

The correspondence in the pernicious anemia, as well as in the chlorosis cases, was close. The carcinomas and sarcomas showed no characteristic difference between the estimated and the v. Fleischl hemoglobin. The three stages of syphilis showed a somewhat higher specific gravity than the percentage of hemoglobin (v. Fleischl) would indicate. In the case of pulmonary tuberculosis with afternoon temperature, the hemoglobin percentage (v. Fleischl) was lower than that estimated from the specific gravity. In the nephritis cases, the difference was slight. In three cases of circulatory disturbance, the hemoglobin per cent. was much lower than that indicated by the specific gravity and in four cases there was practically no difference. In the pregnancy records, the hemoglobin was slightly less than would be indicated by the specific gravity. In the cases of epilepsy, the specific gravity estimated hemoglobin was higher than that shown by the hemometer. In appendicitis, the estimated hemoglobin was lower than that determined by the hemometer; in one case slightly higher. In the remaining cases there was no characteristic difference.

TABLE OF RECORDS.

| Case number. | Specific gravity. | Hemoglobin % estimated from sp. gr. | Hemoglobin % by v. Fleischl's. | Blood Count. | | Sex. | Diagnosis. | Remarks. |
|---|---|---|---|---|---|---|---|---|
| | | | | Reds. | Whites | | | |
| 1 | 1060 | 95 | 95 | ............ | ............ | M | Health. | |
| 2 | 1060 | 95 | 95 | ............ | ............ | " | " | |
| 3 | 1060 | 95 | 90 | ............ | ............ | " | " | |
| 4 | 1060 | 95 | 87 | ............ | ............ | F | " | |
| 5 | 1060 | 95 | 86 | ............ | ............ | M | " | |
| 6 | 1060 | 95 | 85 | ............ | ............ | " | " | |
| 7 | 1060 | 95 | 78 | ............ | ............ | " | " | |
| 8 | 1059 | 91.67 | 91.75 | ............ | ............ | " | " | |
| 9 | 1059 | 91.67 | 90.50 | ............ | ............ | " | " | |
| 10 | 1059 | 91.67 | 90 | ............ | ............ | " | " | |
| 11 | 1059 | 91.67 | 90 | ............ | ............ | " | " | |
| 12 | 1059 | 91.67 | 86 | ............ | ............ | " | " | |
| 13 | 1059 | 91.67 | 86 | ............ | ............ | " | " | |
| 14 | 1058 | 88.34 | 96.5 | ............ | ............ | " | " | |
| 15 | 1058 | 88.34 | 95 | 5,400,000 | ............ | " | " ............................ | Reds—by hematocrit—5,200,000. |

| Case number. | Specific gravity. | Hemoglobin % estimated from sp. gr. | Hemoglobin % by v. Fleischl's. | Blood Count. Reds. | Blood Count. Whites. | Sex. | Diagnosis. | Remarks. |
|---|---|---|---|---|---|---|---|---|
| 16 | 1058 | 88.34 | 94 | ............ | ............ | M | Health. | |
| 17 | 1058 | 88.34 | 90 | ............ | ............ | " | " | |
| 18 | 1058 | 88.34 | 80 | ............ | ............ | F | " | |
| 19 | 1058 | 88.34 | 75 | ............ | ............ | " | " | |
| 20 | 1058 | 88 34 | 74 | ............ | ............ | M | " | |
| 21 | 1057 | 85 | 85 | ............ | ............ | " | " | |
| 22 | 1057 | 85 | 78 | 4,480,000 | 8,400 | F | " ............ | Reds—by hematocrit—4,200,000. |
| 23 | 1057 | 85 | 75 | ............ | ............ | " | " | |
| 24 | 1057 | 85 | 73 | ............ | ............ | " | " | |
| 25 | 1057 | 85 | 66 | 4,480,000 | 9,375 | " | " | |
| 26 | 1056 | 80 | 84 | ............ | ............ | M | " | |
| 27 | 1055 | 75 | 78 | ............ | ............ | F | " | |
| 28 | 1055 | 75 | 70 | ............ | ............ | " | " | |
| 29 | 1055 | 75 | 70 | ............ | ............ | " | " | |
| 30 | 1055 | 75 | 65 | ............ | ............ | " | " | |
| 31 | 1055 | 75 | 63 | ............ | ............ | " | " | |
| 32 | 1051 | 66.33 | 54 | ............ | ............ | | | Examined, Dec. 16, '98, July 20, '99, and Sept. 7, '98. Treat.: Iron, arsenic, strychnine. Improved. |
| 33 | 1054 | 72.5 | 74 | ............ | ............ | " | Simple anemia............ | |
| 34 | 1060 | 95 | 81 | ............ | ............ | | | |
| 35 | 1059 | 92 | 88 | ............ | ............ | " | " " | |
| 36 | 1059 | 92 | 85 | ............ | ............ | " | " " | |
| 37 | 1058 | 88.3 | 83 | ............ | ............ | " | " " | |
| 38 | 1057 | 85 | 90 | ............ | ............ | M | " " | |
| 39 | 1056 | 80 | 75 | ............ | ............ | " | " " | |
| 40 | 1054 | 72.5 | 75 | ............ | ............ | F | " " | |
| 41 | 1054 | 72.5 | 70 | ............ | ............ | " | " " | |
| 42 | 1054 | 72.5 | 70 | ............ | ............ | " | " " | |
| 43 | 1054 | 72.5 | 70 | ............ | ............ | " | " " | |
| 44 | 1054 | 72.5 | 65 | ............ | ............ | " | " " | |
| 45 | 1053 | 70 | 72 | ............ | ............ | " | " " | |
| 46 | 1052 | 67.6 | 70 | ............ | ............ | " | " " | |
| 47 | 1052 | 67.6 | 67 | ............ | ............ | " | " " | |
| 48 | 1051 | 66.3 | 67 | ............ | ............ | " | " " | |
| 49 | 1051 | 66.3 | 50 | 3,600,000<br>3,750,000 | 18,200<br>20,300 | " | Subphrenic abscess. | |
| 50 | 1050 | 65 | 64 | ............ | ............ | " | Simple anemia. | |
| 51 | 1050 | 65 | 63 | ............ | ............ | " | " " | |
| 52 | 1048 | 55 | 35 | ............ | ............ | " | " " | |
| 53 | 1045 | 45 | 66 | 3,450,000 | 31,200 | " | " " | |
| 54 | 1042 | 42 | 45 | ............ | ............ | " | " " | |
| 55 | 1056 | 80 | 80 | ............ | ............ | " | Pernicious anemia. | |
| 56 | 1052 | 67.6 | 60 | ............ | ............ | M | " " | |
| 57 | 1046 | 48 | 33 | ............ | ............ | " | " " | |
| 58 | 1040 | 40 | 40 | ............ | ............ | " | " " | |
| 59 | 1040 | 40 | 35 | ............ | ............ | " | " " | |
| 60 | 1032 | 25 | 33 | 1,344,000 | 6,250 | " | " " | Arsenic, bone marrow, strychnine, cinchona. |
| 61 | 1043 | 43 | 35 | 1,500 000 | 12.500 | " | " " | |
| 62 | 1045 | 45 | 39 | 2,520,000 | 8,000 | " | " " | |
| 63 | 1057 | 85 | 83 | ............ | ............ | F | Chlorosis. Improved. | |
| 64 | 1055 | 75 | 80 | ............ | ............ | " | " " | |
| 65 | 1055 | 75 | 68 | ............ | ............ | " | Chlorosis. | |
| 66 | 1062 | 95 | 85 | ............ | 15,000 | M | Epithelioma of esophagus. | |
| 67 | 1059 | 92.3 | 93 | ............ | ............ | F | Carcinoma—stomach. | |
| 68 | 1054 | 71.6 | 62 | ............ | ............ | " | Sarcomatosis. | |
| 69 | 1043 | 43 | 46 | 2,400,000 | 15,400 | M | " | |
| 70 | 1036 | 31.6 | 40 | ............ | ............ | " | Sarcoma in axilla. | |
| 71 | 1056 | 77.1 | 75 | ............ | ............ | " | Primary syphilis. | |
| 72 | 1060 | 95 | 66 | ............ | ............ | F | Secondary syphilis. | |
| 73 | 1059 | 92.5 | 85 | ............ | ............ | M | " " | |
| 74 | 1058 | 90.1 | 64 | ............ | ............ | F | " " | |
| 75 | 1056 | 77.1 | 78 | ............ | ............ | M | " " | |
| 76 | 1045 | 75 | 65 | ............ | ............ | F | " " | |
| 77 | 1049 | 60 | 45 | 2,900,000 | 21,875 | M | Tertiary syphilis and tuberculosis. | |
| 78 | 1060 | 95 | 68 | ............ | ............ | F | Tuberculous cervical nodes. | |
| 79 | 1058 | 90 | 82 | ............ | ............ | M | Pulmonary tuberculosis..... | Afternoon temp're. Purulent sputum. |
| 80 | 1058 | 90 | 80 | ............ | ............ | F | " " ..... | " " |

TABLE OF RECORDS—(*Continued*).

| Case number. | Specific gravity. | Hemoglobin % estimated from sp. gr. | Hemoglobin % by v. Fleischl's. | Blood Count. Reds. | Blood Count. Whites. | Sex. | Diagnosis. | Remarks. |
|---|---|---|---|---|---|---|---|---|
| 81 | 1057 | 85 | 77 | .............. | ........... | M | Pulmonary tuberculosis..... | Afternoon temp're. Purulent sputum. |
| 82 | 1057 | 85 | 73 | .............. | ........... | " | " " ..... | " " |
| 83 | 1057 | 85 | 66 | .............. | ........... | " | " " ..... | " " |
| 84 | 1056 | 80 | 57 | .............. | ........... | " | " " ..... | " " |
| 85 | 1055 | 75 | 76 | .............. | ........... | " | " " ..... | " " |
| 86 | 1055 | 75 | 73 | .............. | ........... | " | " " ..... | " " |
| 87 | 1054 | 71.6 | 57 | .............. | ........... | " | " " ..... | " " |
| 88 | 1053 | 70 | 54 | .............. | ........... | " | " " ..... | " " |
| 89 | 1052 | 67.5 | 62 | 4,000,000 | 9,375 | F | General tuberculosis. | |
| 90 | 1050 | 65 | 46 | .............. | ........... | M | Pulmonary tuberculosis. | |
| 91 | 1044 | 44 | 56 | .............. | ........... | F | " " | |
| 92 | 1056 | 77.1 | 80 | .............. | ........... | " | Nephritis, chronic interstitial. | |
| 93 | 1056 | 77.1 | 79.5 | 5,400,000 | 6,500 | " | " " | |
| 94 | 1055 | 75 | 75 | .............. | ........... | " | " " | |
| 95 | 1054 | 71.6 | 68 | .............. | ........... | " | " " | |
| 96 | 1039 | 39 | 39 | 1,400,000 | 7,800 | M | Chronic parenchymatous nephritis. | |
| 97 | 1060 | 95 | 92 | .............. | ........... | F | " " | |
| 98 | 1060 | 95 | 91 | .............. | ........... | M | Cardiac asthma. | |
| 99 | 1060 | 95 | 90 | .............. | ........... | " | Valvular disease. | |
| 100 | 1059 | 92.5 | 92 | .............. | ........... | " | " " | |
| 101 | 1058 | 90 | 80 | .............. | ........... | F | Dilatation heart. | |
| 102 | 1054 | 71.6 | 57 | .............. | ........... | M | Aortic insufficiency, convalescent pleurisy with effusion and pericarditis. | |
| 103 | 1046 | 48 | 62 | 5.300,000 | 9,375 | " | Aortic insufficiency,malaria | |
| 104 | 1046 | 48 | 38 | 2,500,000 | 1,500 | " | Valvular lesion. | |
| 105 | 1060 | 95 | 80 | .............. | ........... | F | Pregnancy. | |
| 106 | 1059 | 92.3 | 84 | .............. | ........... | " | " | |
| 107 | 1059 | 92.3 | 75 | 4,000,000 | 18,750 | " | " | |
| 108 | 1058 | 90 | 77 | .............. | ........... | " | " | |
| 109 | 1052 | 67.6 | 61 | 409,600 | 18,750 | " | Post partum hemorrhage. | |
| 110 | 1059 | 92.3 | 81 | .............. | ........... | M | Enteric fever. | |
| 111 | 1056 | 77.1 | 70 | .............. | ........... | " | " " | |
| 112 | 1058 | 90 | 87 | .............. | ........... | " | " " | |
| 113 | 1060 | 95 | 85 | .............. | ........... | F | Epilepsy. | |
| 114 | 1060 | 95 | 80 | .............. | ........... | M | " | |
| 115 | 1058 | 90 | 85 | .............. | ........... | " | " | |
| 116 | 1058 | 90 | 74 | .............. | ...... ... | F | " | |
| 117 | 1057 | 85 | 65 | .............. | ........... | " | " | |
| 118 | 1050 | 65 | 75 | .............. | ........... | " | " | |
| 119 | 1056 | 77.1 | 89 | 5,750,000 | 10,900 | " | Appendicitis. | |
| 120 | 1052 | 67.5 | 86 | 5,500,000 | 12,500 | M | " | |
| 121 | 1050 | 65 | 62 | 2,380,000 | 10.930 | " | " | |
| 122 | 1059 | 92.3 | 90 | .............. | ........... | F | Neurasthenia. | |
| 123 | 1058 | 90 | 90 | .............. | ........... | M | " | |
| 124 | 1057 | 85 | 85 | .............. | ........... | F | " | |
| 125 | 1058 | 90 | 83 | .............. | ........... | M | Senility. | |
| 126 | 1057 | 85 | 78 | .............. | ........... | F | " | |
| 127 | 1060 | 95 | 76 | .............. | 11,560 | M | 2d infection after operation. | Inguinal hernia. |
| 128 | 1052 | 67.5 | 70 | .............. | 13,125 | " | " " " | Femoral hernia. |
| 129 | 1055 | 75 | 68 | .............. | 15,540 | " | Empyema. | |
| 130 | 1054 | 72 | 80 | 5,100,000 | 10,900 | " | " | |
| 131 | 1058 | 90 | 70 | .............. | ........... | F | Chronic suppurative mastitis. | |
| 132 | 1056 | 77.1 | 67 | ...... ...... | ........... | M | Septic thrombosis, vein right leg. | |
| 133 | 1052 | 67.5 | 70 | 4,880,000 | 28,781 | " | Myocitis (suppurative), axilla. | |
| 134 | 1060 | 95 | 92 | .............. | .. ........ | " | Chronic diarrhea. | |
| 135 | 1059 | 92.3 | 90 | .............. | ........... | " | Addison's disease. | |
| 136 | 1058 | 90 | 73 | .............. | ..... ...... | " | Melancholia. | |
| 137 | 1058 | 90 | 84 | .............. | ........... | " | Multiple sclerosis. | |
| 138 | 1058 | 90 | 70 | 5,487,500 | 28,229 | " | Lobar pneumonia. | |
| 139 | 1056 | 77.1 | 77 | 4.337,500 | 11,714 | " | Hodgkin's disease. | |
| 140 | 1055 | 75 | 65 | .............. | ........... | F | Rheumatism (chronic). | |
| 141 | 1050 | 65 | 70 | .............. | 10,625 | M | Gangrene of feet. | |
| 142 | 1047 | 50 | 60 | 3,900,000 | 14,800 | F | Microcephalus. | |

A fair idea of the relation of the specific gravity to the percentage of hemoglobin in the blood is given in Menicanti's conclusions and in those of Dieaballa.

Menicanti[22] says: "In health there is a certain constant relation between the specific gravity of the blood and its percentage of hemoglobin, with very small and varying individual differences. The same holds good in chlorosis, in ordinary anemias and other diseases. In pregnancy and often in heart disease, the relation varies, so that the same percentage of hemoglobin denotes a lower specific gravity."

Dieaballa[8] concludes:

1. The specific gravity of the blood depends, in the first place, upon its hemoglobin contents, but with the same quantity of hemoglobin differences as high as 13.5 per mille specific gravity may occur.

2. 10 per cent. hemoglobin (according to v. Fleischl) denote a specific gravity of 4.46 (according to Hammerschlag.)

3. With the same percentage of hemoglobin, both under physiological and pathological conditions, in women the specific gravity is from 2 to 2.5 lower than in men.

4. The specific gravity varies within wider limits in blood that is rich in hemoglobin, than in blood that is poor in hemoglobin.

5. In nephritis, owing to the hydremia of the plasma, the specific gravity is from 4 to 5 per mille lower than in blood of the same hemoglobin percentage and number of corpuscles in secondary anemia.

6. In leukemia the specific gravity is higher than would be excepted from the percentage of hemoglobin. This difference appears to be in proportion to the greater number of leucocytes.

7. In chlorosis, the specific gravity is about 2.5 per mille higher than in secondary anemia. This difference rapidly disappears during blood regeneration.

8. In those forms of pernicious anemia, where the number of red blood corpuscles is appreciably diminished in relation to the hemaglobin, the specific gravity is about 2 per mille lower than in secondary anemia. During blood regeneration this disappears.

9. The number of red corpuscles in the blood may have a positive influence upon the specific gravity, independently of the hemoglobin, since with the same percentage of hemoglobin differences in specific gravity of 4 to 5 per mille may be produced from this cause.

Our conclusions may be briefly summarised as follows:

1. In most cases the specific gravity and the percentage of hemaglobin of the blood present such a close relation to one another that the latter may be predicted from the former with sufficient accuracy for clinical purposes.

2. Both v. Fleischl's and Gowers's instruments are liable to an error of 10 per cent. or more.

3. There is liable to be very slight if any error in the determination of the specific gravity by Hammerschlag's method.

### BIBLIOGRAPHY.

1. *Arronet:* Maly-Jahresbericht für Thierchemic, 1887.

2. *Becquerel and Rodier:* Recherche sur la Composition de Sang. Deutsch von Eisemann, Erlangen, 1845.

——— Traite de Chimie Pathologique. Paris. 1854, p. 39.

3. *Castellano, P. F.:* Sulla Densita del Sangue, Gazz. d. Osp Milan, 1892, xiii., 718-790.

4. *Copeman, I. M.:* Report on the Specific Gravity of the Blood in Disease. British Med. Journ., London, 1891, i., 161-163.

5. *Dastre, A.:* Methode Nouvelle pour la Determination de la Densite du Sang. Arch. de Physiol., 1893, p. 791; also extract Centralb. für die Med. Wiss., Vol. xxxii., p 461.

6. *Dehio, K:* Zur Kritik des Fleischlchen Hämometer. Verhandl. d. Cong. f. Innere Med., Wiesb., 1892, xi., ii., 135-143

7. *De Voto, L:* Ueber die Dichte des Blutes unter Pathologischen Verhältnissen. Ztschr. f. Heilk., Berlin, 1890, xi, 175-186.

8. *Dieaballa:* Deutsches Archiv. f. Klin. Med., 1896, Bd. 57.

9. *Eijkman:* Bluntuntersuchungen in den Tropen, Virchow's Archiv., Bd. 126, 1896, p. 113.

10. *Fano, G.:* Di una nuova funzionei de corpuscoli rossi de Sangue Sperimentale. Firluze, 1882, i, 256, 370.

11. *Glogner:* Specifische Gewicht des Blutes in den Tropen, Virchow's Archiv., Vol. 126, p. 109.

12. *Hammerschlag, A:* Ueber das Verhalten des Specifischen Gewichts des Blutes in Krankheiten. Centralblt. für Klin Med, Leipzig, 1891, xii., 825-837.

13. ——— Eine Neue Methode zur Bestimmung des Specifischen Gewichts des Blutes. Ztschr f. klin. Med, Berlin, 1892, xx., 444-456.

14. *Koch, A. und Schlesinger, H:* Hämatologische Studien. Beitr. z. Kinderk. a. d. I. off Kinder Rr. Qust. in Wien, 1892, n. F. ii, 1-62.

15. *Hoppe-Seyler:* Beiträge zur Kenntniss der Eigenschaften der Blutfarbstoffe. Ztschr. f. Physiol Chem., Strassburg, 1889 xii, 477-496.

16. *Jones, E L:* On Variations in the Specific Gravity of the Blood in Health. Journ. of Physiol., Cambridge, 1887.

17 ——— Further Observations on the Specific Gravity or the Blood in Health. Jour. of Physiol., Cambridge, 1891, xii, 299-346, 4 pl

18. *Landois and Stirling, W:* Text-book of Human Physiology Translated from 7th German edition, London, 1891, 4th edition

19. *Limbeck v. R. R.:* Grundriss Einer klinischen Pathologie des Blutes. Jena, 1892. Verlag von Gustav Fischer.

20. *Lyonnet B.:* De la Densiti du Sang, la Determination Clinique ses Veriations Physiologiques et Pathologiques. Paris. 1892.

21. *Lichty, Cora:* Philadelphia Med. Jour., 1898, ii., 242-44.

22. *Menicanti:* Ueber das Specifische Gewicht des Blutes und dessen Beziehung zum Hemoglobingehalt. Deutsches Arch. f. klin Med., Leipzig, 1882, i., 407-422.

23. ——— Ueber das Specifische Gewicht des Blutes und dessen Beziehung zum Hemoglobingehalt. Arb. a. d. med. Klin. Inst. d. Univer. zu München, Leipzig, 1893, iii., 490-503.

24. *Monti:* Veränderungen der Blutdichte bei Kindern. Abst. Schmidt's Jahr., Vol. 250, p. 189.

25. *Peiper:* Das Specifische Gewicht. des Menschlichen Blutes. Centrabl. f. Klin. Med., 1891, No. 12.

26. *Roy, C. S.:* Note on a Method of Measuring the Specific Gravity of the Blood for Clinical Use. Edinburgh Clin and Path. Journ., 1883-4, i., 561-563.

27. *Schmaltz, R.:* Das Verhalten des Specifischen Gewichts des Blutes bei Kranken Deutsche Med. Wochenschr., Leipzig, 1891, vxii., 555-559.

28. ——— Die Untersuchung des Specifischen Gewichts des Menschlichen Blutes und das Verhalten Desselben bei Krankheiten. Verhand. d Cong. Innere Med., Wiesb., 1891, x., 427-440.

29. *Siegel, C T.:* Ueber eine Verbesserung der Royschen Methode zur Blutdichtebestimmung und damit Angestellte Untersuchungen bei Kindern. Prag Med. Wochenschr., 1892, xvii., 209, 222, 235.

30. *Sherrington and Copeman:* Variations Experimentally Produced in the Specific Gravity of the Blood. Journ. of Physiol., Cambridge, 1893 xiv., 52-96.

31. *Von Jaksch*: Klinische Diagnostik. Third revised edition. Wien und Leipzig, 1892.

32. *Yarrow and Hitchens:* Clinical Estimation of the Quantity of Hemoglobin in Human Blood. Univ. Med. Mag., xii., 1 p., 26, Oct.

33. *Ziegelroth:* Einfluss des Aderlasses auf das Specifische Gewicht des Blutes. Virchow's Archiv., Bd. 141, p. 395. 1895.

34. ——— Das Specifische Gewicht des Menschlichen Körpers und Blutes; Zugleich ein Beitrag zur Krassenlehre Virchow's Archiv., cxlvi., 3, 1896, p. 453.

145 ALLEN STREET.

